# Single molecule multiplexed nanopore protein screening in human serum using aptamer modified DNA carriers

**DOI:** 10.1038/s41467-017-01584-3

**Published:** 2017-11-16

**Authors:** Jasmine Y. Y. Sze, Aleksandar P. Ivanov, Anthony E. G. Cass, Joshua B. Edel

**Affiliations:** Department of Chemistry, Imperial College London, Exhibition Road, London, SW7 2AZ UK

## Abstract

The capability to screen a range of proteins at the single-molecule level with enhanced selectivity in biological fluids has been in part a driving force in developing future diagnostic and therapeutic strategies. The combination of nanopore sensing and nucleic acid aptamer recognition comes close to this ideal due to the ease of multiplexing, without the need for expensive labelling methods or extensive sample pre-treatment. Here, we demonstrate a fully flexible, scalable and low-cost detection platform to sense multiple protein targets simultaneously by grafting specific sequences along the backbone of a double-stranded DNA carrier. Protein bound to the aptamer produces unique ionic current signatures which facilitates accurate target recognition. This powerful approach allows us to differentiate individual protein sizes via characteristic changes in the sub-peak current. Furthermore, we show that by using DNA carriers it is possible to perform single-molecule screening in human serum at ultra-low protein concentrations.

## Abstract (web summary)

It is still a challenge for current nanopore sensing methods to differentiate multiple analytes from complex biological material. Here, the authors graft nucleic acid aptamer sequences along the backbone of a double stranded DNA carrier for the detection of multiple protein targets in human serum.

## Introduction

Measurable changes in the concentration of proteins found in serum as well as other physiological fluids can often be indicative of disease; with early diagnosis allowing the implementation of accurate and effective treatment to prevent disease progression. While many different biosensors have been demonstrated with the ability to identify and quantify proteins, they usually lack either the specificity and/or sensitivity for early stage diagnosis in real samples containing many (thousands) of background proteins, often at concentrations orders of magnitude higher than the target markers. Nanopores^1–4^ have been proven to be a promising tool for the detection of DNA^5–14^, RNA^15, 16^, proteins^17–24^, DNA binding proteins^25–29^ and other molecules^15, 16, 30–32^ as they allow for single molecule level and rare event analysis which is normally masked by ensemble averaging. The detection method is straightforward; as each analyte molecule passes through the pore under the influence of an applied potential—single molecules are identified by transient modulations of the ionic (nanopore) current. These current modulations generally depend on the volume, charge and conformation of a single molecule in the pore. However, detection of proteins using nanopores is often challenging due to their heterogeneous charge, fast translocation times, poor signal-to-noise ratio especially for small proteins (<15 kDa), and non-specific adsorption to the pore. Although improvements have been made, for example, in the use of high bandwidth amplifiers^20, 33^, perhaps the biggest limitation of nanopore protein sensing is in the lack of specificity, making it challenging to differentiate between multiple analytes let alone trying to obtain meaningful data from complex biological matrices such as serum or cerebral spinal fluid.

To date, several strategies have been used to develop solutions to such fundamental problems. For example, Yusko and co-workers enhanced the selectivity through optimisation of the pore surface and the incorporation of mobile lipid bilayer ligands to allow for the detection of amyloid-beta oligomers and fibrils. Further interesting work has allowed quantification of the shape, volume, charge, rotational diffusion coefficient and dipole moment of individual proteins^19, 34^. Alternatively, Singer et al.^35, 36^ presented strategies to detect PNA bound to the large DNA molecule in the process increasing the signal-to-noise ratio of the sub-level ionic current caused by the complex passing through the pore. The ability to detect patterns of protein natively bound to DNA with high concentration of RecA to ensure complete coating of the DNA was also presented^37, 38^. Recently, Bell and co-workers demonstrated an innovative method by using a DNA construct, employing a 7.2 kb single-stranded DNA (ssDNA) as a carrier with 190 oligonucleotides attached. Modification of some of these oligonucleotides with a barcode system created divalent antigen sites on a DNA sequence allowing detection of antibodies^39–41^. The detection system has been shown to detect Biotin, Bromodeoxyuridine, Puromycin and Digoxigenin in a mixed target system. Although exceptionally valuable, these approaches still require extensive engineering, complex immobilisation or conjugation steps, relatively high analyte concentrations, and are restricted by selectivity and non-specific binding. In addition, nanopore detection is often performed at high ionic strengths (up to 4 M LiCl), where the buffer ionic strength and composition significantly deviate from physiological salt concentrations, and can have a dramatic effect on protein binding and conformation.

**Fig. 1:**
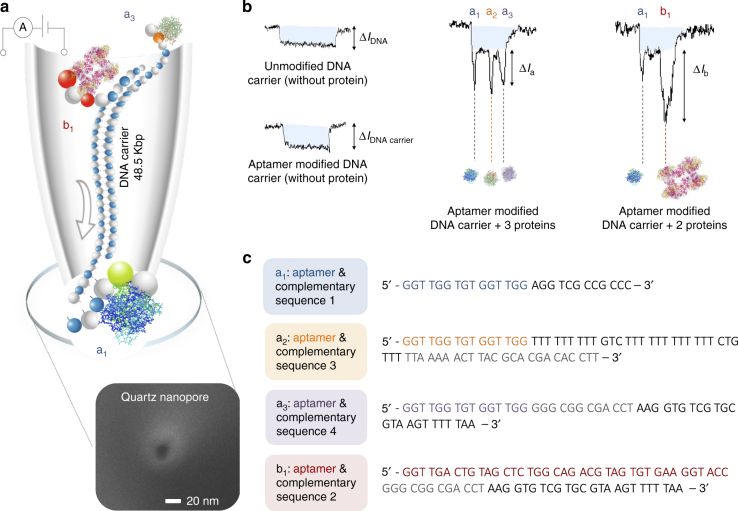
Design concept for using DNA aptamers and a carrier for protein sensing. **a** Schematic representation of a DNA carrier (48.5 kbp) engineered to contain aptamer sequences (a_1_, b_1_, a_3_) that bind to three proteins translocating through a nanopore driven by the electric field. Translocations take place from inside the nanopipette to the outside. As an example, a SEM is shown of a typical nanopipette tip used with a pore diameter of 16 nm. Examples of translocation events of unmodified and aptamer modified carriers are shown in **b**. Also shown are example translocations for the detection of 3 and 2 proteins bound to the aptamers on the DNA carrier. Importantly, the sub-levels can be used to differentiate between proteins both in terms of location and magnitude. **c** Aptamer sequences used for the detection of thrombin (a_1_, a_2_, a_3_) and AChE (b_1_)

To address these limitations, we report on a flexible, selective and generic approach for the accurate detection of multiple proteins with nanopores by utilising nucleic acid aptamers grafted to a double-stranded DNA (dsDNA) molecular carrier, Fig. 1a. Aptamers, ssDNA or ssRNA oligonucleotides, have the ability to non-covalently bind to their target molecule with high affinity and selectivity. Sequences can be selected in vitro by SELEX (systematic evolution of ligands by exponential enrichment) with little to no inconsistency between batches compared with antibodies. Over the last several years, aptamers have been widely used in diagnostics and as therapeutics agents. Importantly, extensions to the aptamer sequence can easily be designed to hybridise to a complementary sequence on a DNA carrier, significantly simplifying the binding chemistry needed for carrier synthesis. We show that it is possible to use λ-DNA terminal overhangs as an ideal attachment site for the aptamer sequence leading to the formation of specific detection probes. The dsDNA act as a generic carrier and the aptamer can be selected to bind/unbind to the carrier at will, while overcoming transport limitations arising from the heterogeneous surface charges of the protein. The presence or absence of a specific target in solution can be identified by quantifying the characteristic transient change in current within the dsDNA level, see examples in Fig. 1b. As the charge of the complex is dominated by the negative charge of the DNA carrier backbone, this method facilitates the efficient transport of net positive/negative or even neutral proteins without modification of the measurement conditions and without the need for sample pre-processing or purification, allowing direct detection at near physiological salt concentrations and as demonstrated here, in complex biological samples.

## Results

### DNA carrier design and nanopore sensing studies

Nanopore experiments were performed using 18 quartz nanopipettes with nanopore diameters of 16 ± 4 nm (the error is defined as 1 standard deviation) as measured using a scaning electron microscope (SEM) for *n* = 4, (e.g., Fig. 1a inset) and a corresponding conductance of 2.8 ± 0.5 nS for *n* = 18 at 0.1 M KCl unless otherwise stated. Electrical and optical characterisation of the pipettes are shown in Supplementary Figs. 1 and 2 respectively. Single nanopores formed at the tip of nanopipette by laser assisted pulling, was our primary choice, due to their straightforward fabrication, relatively low noise characteristics and ease of manipulation with exceptionally high aspect ratio. From SEM and transmission electron microscopy (TEM) characterisation the tip cone angle was measured to be 14–18° over the first 100 nm. The effective length of the nanopore can thus be approximated to be the distance at which 75–80% of the total resistance drops which corresponds to an effective length of ~45 nm. The analyte was introduced inside the pipette together with the patch/bath electrode, and a ground/reference electrode outside the pipette. In this configuration, under applied negative voltage, translocations occur from inside of the nanopipette (cis) to the outside (trans) resulting in an overall increase in the conductance when using 0.1 M KCl^14^.

dsDNA is an ideal molecular carrier, due to its rigidity and nominal sensing times especially in comparison to ssDNA^39–41^. In this work specifically, λ-DNA was selected as a carrier due to the well-defined current signature as well as the presence of 12 base overhangs on both the 5′ and 3′ termini enabling hybridisation of a complementary oligonucleotide. The complementary oligonucleotide was further extended with aptamer sequences leading to the formation of a specific detection probe to target either thrombin or acetylcholinesterase (AChE), Fig. 1c, Supplementary Figs. 3 and 4. The initial 27-mer design (15 aptamer sequence + 12 base overhang) allowed the rapid and distinct detection of two targets as seen by two peaks on either side of the signatures. To evaluate the specificity and suitability of the detection probes, we chose to detect the same targets on both ends of the carrier before moving to different targets system.

**Fig. 2:**
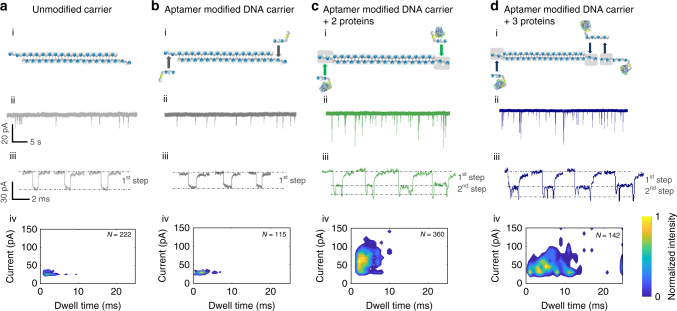
Current-time trace and statistics for sensing proteins bound to a modified carrier. Translocation and statistics for **a** 100 pM of unmodified λ-DNA, **b** 100 pM aptamer modified λ-DNA (1:1 ratio after filtration), **c** 100 pM aptamer modified λ-DNA with two probes for the detection of thrombin (1.6 nM each), **d** 100 pM aptamer modified λ-DNA with three probes for the detection of three thrombin targets (1.6 nM each). All experiments were performed in 100 mM KCl and at a voltage of −200 mV, taken using 4 different nanopipettes for each sample and re-filtered to 5 kHz. Typical individual translocation events are shown with the contribution due to the DNA carrier labelled as “1st step” and the protein contribution labelled as “2nd step”. Scatter plots shown in (iv) clearly indicate binding of protein due to the substantial increase in translocation times and current blockades and corresponding sub-peaks as shown in (iii)

Control experiments were performed with unmodified λ-DNA before and after aptamer hybridisation, Fig. 2a, b. The mean current signature and dwell times for unmodified carrier and aptamer modified carrier were found to be (29.4 ± 2.6, 29.8 ± 5.4) pA and (3.0 ± 1.7, 4.3 ± 2.1) ms respectively at an applied potential of −200 mV which is consistent with previously published reports for a similar nanopore system^42^. A scatter plot analysis revealed very similar sensing signatures and negligible change in the current and dwell time distributions when comparing with aptamer modified and unmodified DNA carrier, Fig. 2a, b. Additional scatter plots at two different voltages (−180 and −200 mV) for both aptamer modified and unmodified DNA carrier are available in Supplementary Fig. 5.

The protein target was human alpha thrombin (α-thrombin) (M.W. 37.5 kDa; pI of 7.0–7.4), a trypsin like serine protease, that is the only enzyme capable of catalysing the conversion of soluble fibrinogen into insoluble fibrin. An elevated pM range of thrombin in blood has been linked to thrombotic diseases, and for early stage detection of thrombosis, it is essential to detect this protein at trace levels with high sensitivity. The corresponding aptamer is a thrombin-binding aptamer (TBA), a 15-mer (5′-GGTTGGTGTGGTTGG-3′) with a well-studied G-quadruplex structure^43, 44^, that binds α-thrombin selectively with a *K*_d_ ~ 35–100 nM in solid phase assays^45^. Prior to nanopore measurements, the hybridisation of the aptamer probes to the λ-DNA carrier was confirmed by agarose gel electrophoresis, Supplementary Fig. 6. A gel shift assay showed the complex consists of thrombin and TBA with a retardation expected for protein bound to the aptamer, refer to Supplementary Fig. 7. Further experimental controls were performed by hybridising non-specific oligonucleotides to the dsDNA followed by incubation with thrombin. For these samples, individual current signatures with mean sensing times (3.1 ± 1.5 ms) were recorded, which were comparable to those of unmodified λ-DNA and modified carriers without protein, Supplementary Fig. 8.

Addition of 1.6 nM thrombin (ratio being ~1:1 between protein and DNA) resulted in binding of the protein to the TBA attached to the DNA carrier and subsequent nanopore detection of the bound protein. It should be noted that detection of such low protein concentrations is not typical when sensing proteins natively without a carrier, due to fast translocation times and event rates significantly lower than predicted from the Smoluchowski rate equation, which often necessitate protein concentrations well in excess of 10s-100s nM^17^.

The detected events revealed a distinct 2-step signature for thrombin bound to the DNA carrier, Fig. 2c, d (iii). The 1st step, was associated with the DNA carrier and had an overall increase in conductance with a mean dwell time comparable with unmodified λ-DNA and modified carriers without protein. A 2nd step occurred at the beginning and ends of the events, corresponding to the thrombin binding to the detection probe with a 1.8x increase in the total current compared to the carrier. This two-step signature was attributed to the negatively charged detection probe (aptamer plus complementary sequence) and presence of negatively charged residues on the surface of bound thrombin that at pH 8 enhanced further the ion flow. Although the majority of events (~83%) exhibited a two-step signature with similar overall amplitudes, a small fraction (~5%) exhibited much larger peak current and slower detection times, Fig. 2c (iv). For example, compared to unmodified λ-DNA and aptamer modified λ-DNA the most probable dwell time was 1.4 ms longer and is attributed to possible conformation changes due to TBA binding to thrombin. X-ray crystal structure reveals TBA folds into two G-quartets connected by two TT loops upon interaction with the thrombin anion exosite I, while TGT loop is in close proximity to the heparin binding site of neighbouring thrombin molecules giving dimensions of 6 × 12 × 6 nm^43, 46^. In addition, the presence of high concentration potassium ions likely enhanced the formation of TBA- thrombin complexes as potassium ions stabilise the G-quadruplex structure^47^.

### Sensing of the DNA carrier with up to three targets proteins

The DNA carrier could further be extended to incorporate a third aptamer sequence. This was engineered by extending the second binding domain by 51 bases. Hybridisation of the complementary part of the second binding domain with an additional 30-mer oligonucleotide aptamer allowed the formation of a third target binding region. Due to potential steric interactions, the second and third aptamer sequences were designed on the opposite strands of the carrier to provide adequate spatial separation upon aptamer binding to the protein target. In the first instance, TBA was used at all three binding sites and the carrier was initially incubated with three-fold excess of thrombin for 45 min prior to performing the experiments.

Analysis of the current-time trace again showed a two-step signature, however with 3 distinct peaks, corresponding to each bound protein target, superimposed on the carrier signal. One of the peaks was located close to the tail end of the molecule and the other two peaks were in close proximity to each other. We believe observation of distinguishable peaks with proteins in close proximity is due to a combinational of the geometry of the nanpopore, the size of the protein TBA complex being close to that of the number, balance between electro-osmotic and electrophoretic forces and the electrolyte concentrations we use. The measured current signatures were consistent with the sequence design where the two binding sites were designed to be in close proximity, spaced 66 bases (~19.8 nm) apart. Based on the current signature it was possible to correlate a rational insertion direction of the carrier into the nanopore via the 5′ (43%) and 3′ (57%) ends. The current amplitude of the 2nd step was consistent with experimental finding for a carrier with two TBA’s bound as shown in Fig. 2c, and confirmed that the extended aptamer probe is not affected by the extra binding domain. It should be noted that a small fraction of events (9%) with event times below 500 μs was excluded from the data analysis as these were attributed to the detection of unbound thrombin in solution. Scatter plot analysis revealed substantial differences between the current and dwell time distributions when comparing unmodified/aptamer modified DNA carrier with the carrier complex bound to two or three protein targets, Fig. 2 (iv). With three targets bound, substantial broadening of the dwell time distribution was observed and was likely due to a greater interaction between the nanopore walls and the protein/DNA carrier transported from the inside of the nanopipette to the outside.

At first glance, it is somewhat surprising that the 3rd peak was clearly identifiable in individual events, considering that the spatial separation between the 2nd and 3rd binding sites, including aptamers was 96 bases or ~29 nm, with an average translocation velocity of ~8 mm/s. However, it should be stressed that generally with bulky, less-charged complex bound, such as the proteins used here, the velocity is not constant across the full molecular length. This is in part due to a larger entropic barrier needed to thread the protein segments of the modified DNA into the nanopore. A similar effect has been observed by Singer et al.^35^. This in turn will result in additional times required for threading the bound analytes. The latter is indicated in our data whereby the signal arising from the protein take place over longer timescales than typically expected leading to broader dwell time distributions, as can be seen in the scatter plots in Fig. 2c, d.

The potential to extend the DNA carrier further on either the 5′ or 3′ ends of λ-DNA could lead to even more binding domains, certainly providing an accurate and flexible route to detection of multiple protein targets simultaneously. While we could clearly discriminate analytes which are 96 bp apart the upper limit of how many analytes could be multiplexed per carrier should be approached with caution as the sensing method is a stochastic drift–diffusion process and there is a spread of the translocation times in successive peaks with bound analytes as observed by Bell et al.^40^.

However, assuming a binding domain every 1000 bases (~300 nm), this may allow for >40 protein targets to be detected. Previously, it has been shown that ssDNA carriers could be modified with antigens in order to detect antibodies^39, 40^ however, this requires conjugation chemistry and depends on how efficiently this process occurs. More importantly, due to the size of the antigen and antibody it will likely be hard to differentiate between bound protein and potential changes in the confirmation of the antigen (e.g. folded vs unfolded). Whereas our approach is solely reliant on DNA hybridisation chemistry and the interaction between short aptamer sequences (<50 nucleotides) and protein. As shown, the aptamer bound to the carrier on its own does not contribute to the background signal.

**Fig. 3:**
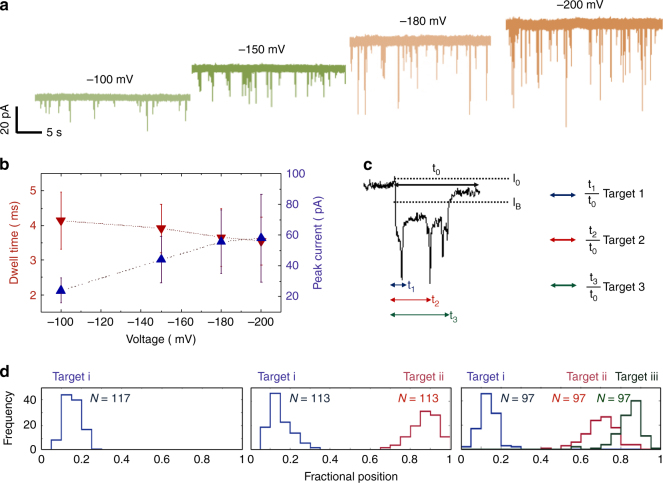
DNA carrier with 3 aptamer targets. In all cases a concentration of 100 pM in 100 mM KCl was used at an applied voltage ranging between −100 to −200 mV. **a** Typical voltage dependant current-time trace, re-filtered to 5 kHz, is shown. **b** Voltage-dependent relationship between the dwell time and peak current: increasing voltage lead to electrophoretic force exerted on the dsDNA backbone leading to small decrease in dwell time and higher peak currents. **c** A typical translocation event with three distinct sub-peaks is shown with a description of how the fraction position (i.e., relative location) of the bound protein is determined. **d** As can be clearly seen, it is possible to differentiate between DNA carriers with 1, 2 and 3 protein targets attached. *N* = total number of protein events within the same carrier

Voltage dependence for three targets on the DNA carrier signature is shown in Fig. 3a with applied potentials of −100, −150, −180 and −200 mV respectively with mean dwell times and peak currents summarised in Fig. 3b. As shown, expected trends are observed, whereby increasing the applied voltage leads to larger peak current (from 44.0 ± 14.9 to 59.2 ± 24.3 pA) and a moderate decrease in dwell times (from 3.9 ± 0.7 to 3.6 ± 0.7 ms) from −150 to −200 mV. This trend is consistent with the predominantly electrophoretic force exerted on the negatively charged dsDNA backbone rather than individual bound targets in the electrolyte at pH 8.

It is important to note that false positives may arise due to single and multiple knots being observed in long DNA strands such as the carrier used here^48^. However, under our experimental conditions the observations of DNA knots were very rare (<1%), see Supplementary Table 1. This was likely due to the electrolyte composition and low ionic strength used (0.1 M KCl), low pass filtering cut-off (frequency 5–10 kHz) and relatively fast knot transit time. Under these conditions it is likely any knots would be embedded in the noise and have negligible effect on the discrimination of targets bound to the DNA carrier. Furthermore, partially folded DNA would potentially result in more complex ionic current blockades whereby the protein signal would not necessarily appear at the front or end of the DNA carrier.

To demonstrate that nanopores can not only count the targets bound to its specific aptamer probes, but can also be used to accurately determine the location between different aptamer probes/targets along a DNA molecule the sensing times were normalised with 0 being defined as the onset and 1 being defined as the end of the event. This is needed to take into account the differences in dwell times so that the fractional position of the bound protein to the DNA carrier can be determined, Fig. 3c. This is carried out by dividing the times associated with the position of target 1, 2 and 3 (*τ*_1_, *τ*_2_ and *τ*_3_) and dividing by the total duration (*τ*_0_). As shown in Fig. 3d, the signal could be broken down into sub-populations consisting of 1, 2 and 3 proteins bound to the same DNA carrier labelled target i, ii and iii with a total of 117, 113 and 97 events. Although we were able to detect different targets along the same DNA carrier, we observed 17% less events in the third binding site when comparing to binding 1 or 2 targets, this is due to the binding domains is dependent on hybridising to the second binding domain. This could easily be overcome by ligation of the relevant sequences in future experiments. In all cases the fractional position corresponded well to the spacing between the different targets. Conversely, the same approach can in principle be used for the identification and positional mapping of specific sequence motifs in a carrier with unknown sequence.

### Sensing of multiple protein targets

**Fig. 4:**
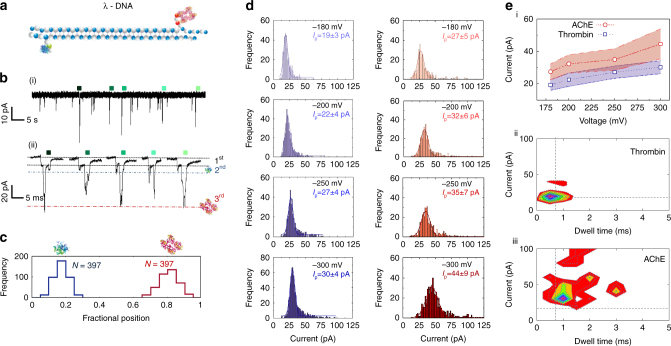
Nanopore sensing of multiple protein targets on a single DNA carrier. **a** 2D schematic of the λ-DNA carrier with two independent aptamer probes specific to thrombin (dimensions: 87.7 × 67.8 × 61.1 Å) and AChE (dimensions: ~211.6 × 129.7 × 195.4 Å). **b** Typical current-time trace recorded at −200 mV and re-filtered at 5 kHz clearly showing three levels. The 1st associated with the DNA carrier, 2nd with thrombin (~16–34 pA) and 3rd from AChE (~22–57 pA). **c** Only the translocation events exhibiting two distinct sub-peak were used for further analysis. As can be seen the fractional position correlates well with the relative positions of thrombin (0.2) and AChE (0.8). **d** Individual sub-peak amplitude and dwell time statistics obtained at voltages ranging from −180 to −300 mV. Values after ‘±’ represent one standard deviation. The mean peak current (*n* = 397) was determined via Gaussian fitting and summarised in **e** (**i**). Importantly both proteins are easily distinguishable not only by location but also by the current amplitude and dwell time, as observed by the individual scatter plots of the sub-peaks as shown in **e** (**ii**) and **e** (**iii**) for thrombin and AChE, respectively

Our detection strategy can not only determine differences in sub-peak position and hence protein location but can also be used to differentiate between size simply based on the peak magnitude of the ionic current. To demonstrate the generality of our approach, we carried out simultaneous sensing of different protein targets with a single carrier. TBA was hybridised to the 3′ end of the opposite strand whilst a different, 39-mer aptamer sequence selective for AChE was hybridised to the 3′ end (5′-GGTTGACTGTAGCTCTGGCAGACGTAGTGTGAAGGTACC-3′), Fig. 4a. AChE (280 kDa, pl of 5.0–5.3), is a key enzyme at the neuromuscular junction in controlling the concentration of neurotransmitter, acetylcholine in mammals. AChE was an ideal point for comparison to thrombin due to its substantially larger molecular size (280 kDa vs. 37.5 kDa) and a reported recognition aptamer sequence with high affinity (*K*_d_ = 14 ± 1 pM) at an ionic strength comparable to our experimental conditions^49, 50^.

To ensure successful experiments and minimise any blockage, slightly larger pores with an average diameter of 35 ± 5 nm were used (see Supplementary Fig. 1b for electrical characterisation of 20 quartz nanopores). It should be noted that no events were observed at −100 mV indicating higher electrophoretic forces are required to allow the molecule pass through as there is a possibility of electro-osmotic forces acting on the complex when placed inside the negatively charged quartz pipette. Representative ionic current traces and expanded insets are shown in Fig. 4b for a DNA carrier concentration of 100 pM and an applied bias of −200 mV. The events clearly show three levels with the lowest being associated with the DNA carrier, a second level corresponding to thrombin bound to TBA on the carrier, and finally the largest current step corresponding to AChE bound to its respective aptamer, scatter plots can be found in Supplementary Fig. 9. Control experiments, performed with a mixture of modified and unmodified lambda DNA are shown in Supplementary Fig. 10. Interestingly the majority of events (~73%) were insertions via the 3′ end first, which is likely due to the relative size and spherical shape of thrombin being easier to pass through the nanopore. The relative order of magnitude of the sub-peaks correlate well to the relative sizes and hence excluded volumes of the individual proteins. For example, the volume of a protein can be approximated to be equal to *V* (nm^3^) = 1.212 × 10^−3^ (nm^3^/Da) × Ma (Da), for thrombin and AChE this gives 45 nm^3^ and 339 nm^3^ respectively. Therefore, AChE is ~7.5x larger and therefore has a larger excluded volume as can be seen from the 1.5–3x increase in current blockade. As a first approximation, the change in current is proportional to the excluded volume^51^; however, the absolute value cannot be calculated without first knowing *H*_eff_ (effective height of nanopore) and *f* (geometrical correction factor). Although it can be argued that AChE is sufficiently large to be detected without a DNA carrier, the net charge of the protein is low making it difficult to control the transport through the pore. Furthermore, when the protein is bound to the carrier, transport is significantly slower when compared to the free protein in the pore and allowing for improved statistics. The same method is used as in Fig. 3c to discriminate between the two different protein targets. In this case, the majority of events observed had both proteins bound to the carrier with the fractional position being 0.2 and 0.8 for thrombin and AChE respectively, Fig. 4c.

To gain a better understanding of the sub-populations, we further analysed the peak amplitudes as a function of applied voltage for (−180, −200, −250 and −300 mV) for the individual bound proteins to the carrier, Fig. 4. First, increasing the applied voltage leads to a small increase in mean peak current (from 19 ± 3, 27 ± 5 to 30 ± 4, 44 ± 9 pA for thrombin and for AChE. Figure 4e, summarise the voltage dependence of these sub-peak current signatures, while scatter plots of the sub-peaks show it is possible to distinguish between different proteins. It should be noted that on average 10% of all events were observed to have a folded component (as measured for the DNA carrier without protein present), Supplementary Fig. 11 However, the percentage of false positives are even lower as we use a peak current/dwell time threshold to minimise false positives even further. For example, a comparison between sub-peak statistics for the signal attributed to folded DNA, thrombin, and AChE respectively (taken at −200 mV) is shown in Supplementary Fig. 12 and Fig. 4e (ii),(iii) There is <1% overlap between AChE and folded DNA based on the sub-peak amplitude and dwell time and <10% overlap between thrombin and folded DNA. It is expected that the number of false positives would be even lower as there is <3% overlap between the fractional position of a folded event and the corresponding 1st binding site.

### Sensing of protein targets in human serum

**Fig. 5:**
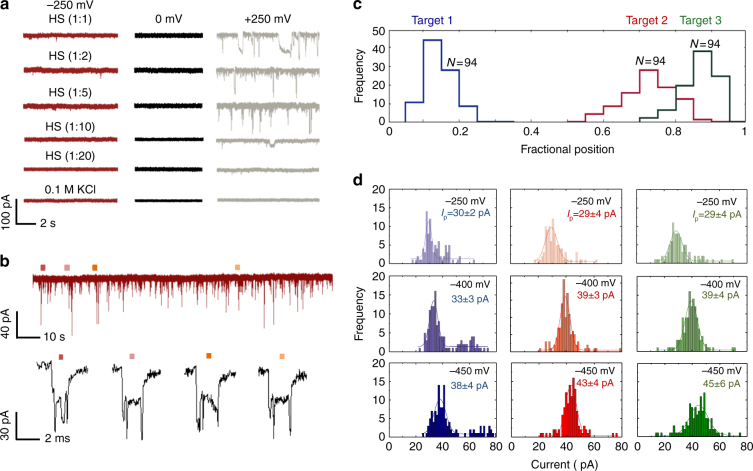
Sensing of protein targets in human serum. **a** Typical current-time traces are shown for a serial dilution of human serum spiked with 100 mM KCl at applied voltages of −250, 0 and +250 mV. In all cases the serum was inserted inside the nanopipette. As is shown a dilution of 1:20 consists of a sufficiently stable baseline to be used in nanopore sensing. **b** DNA carrier with 3 TBA probes were incubated in HS with the current-time traces clearly showing sub-peaks associated with thrombin binding at −250 mV. **c** Similar to Fig. 3, the sub-peaks could easily be distinguished based on location (0.2), (0.7) and (0.9) (*N* = 94). **d** Individual current amplitudes for all three sub-peaks, obtained at an applied voltage of −250, −400 and −450 mV with the average current determined via Gaussian fitting. Values after “±” represent 1 standard deviation

To date, the majority of application relying on nanopore sensing use purified samples, there is therefore a need to develop strategies whereby such detection modalities can be used with unprocessed biological samples. For example, human serum (HS) can have thousands of different proteins, often at much higher concentrations than the target analyte making detection exceptionally challenging. As shown in Fig. 5, the proposed strategy can be used to selectively sense for target proteins in a complex environment. To confirm feasibility a serial dilution of HS with 100 mM KCl was performed at voltages ranging between −250 to +250 mV. This was used to determine the dilution factor necessary to ensure a stable baseline in the ionic current, Fig. 5a. It was found that at both positive and negative bias the optimal dilution was 1:20 (1 μl of HS in 19 μl buffer). In all cases HS was inserted in the nanopippette whilst the other reservoir consists of buffer only. The DNA carrier consisting of 3 TBA aptamers was incubated at this HS concentration for 45 min (sequences used can be found in Supplementary Fig. 4. Ionic current signatures were observed with clearly defined sub-peaks as shown in Fig. 5b, while control experiments with unmodified lambda DNA in serum showed signatures consistent with the ones obtained from unmodified lambda DNA in buffer, Supplementary Fig. 13. For the modified carrier, normalised fractional positions of the sub-peaks were observed at 0.2, 0.7 and 0.9 correlating well to the positions of the 3 aptamer targets and in very good agreement with the measurements performed in buffer solutions (as shown Fig. 5c vs. Fig. 3d).

Target 1, 2 and 3 have a comparable peak centre of 30 ± 2, 29 ± 4 and 29 ± 4 pA respectively at −250 mV and at other applied voltages at −400 and −450 mV, see Fig. 5d. This correlates very well in terms of the target size. Surprisingly, the number of events decreased with increasing applied voltage which may be attributed to possible crowding near the nanopore of background proteins at higher potentials. The contour plot of the mean dwell time with peak current of the whole complex at different applied voltages can also be found in Supplementary Fig. 14. This shows a similar trend to the buffer case where an increase in voltage led to a small decrease in dwell time and larger peak current confirming the detection probes were very specific in the discrimination of the analyte in a complex environment in HS.

## Discussion

In a standard DNA translocation process, electrophoretic forces mainly dominant and the translocation events will have both folded and unfolded events. It can also be assumed the velocity of the translocation will be relatively constant. However, when small protrusions are added to the DNA, as demonstrated by Plesa et al.^52^ electrophoretic forces still dominate, the molecule travels slower even with symmetric construct indicating an existence of non-linear translocation process. This along with the highly confined space in the nanopipette, size of protein and aptamer, electrolyte concentration results in a significant slowdown of the molecule, as shown in Fig. 2 as the number of proteins per DNA carrier are increased. The translocation velocity is not constant across the full molecule length and depends on the location as well as the number of proteins attached to the molecule. Perhaps surprisingly, our design allow us detect proteins with ~96 bp spacing. This appears to be competitive and comparable to alternative single-molecule techniques such as super-resolution microscopy^53^.

In summary, we have presented a fully flexible and efficient nanopore sensing method, with single-molecule resolution, that is able to selectively detect multiple proteins via grafting the aptamer sequence into the backbone of a DNA carrier. In comparison to conventional immunoassays or existing multiplexed nanopore method^39–41^, our platform has been able to successfully isolate and identify targets without the need for repeated wash steps, expensive oligomer modification or using high reported concentration of incubated antibodies^39–41^, hence significantly reducing the operation time and the cost. Aptamers are small, highly negatively charged and can be engineered to enhance their target selectivity and binding affinity (typically from nM to pM), allowing further reduction in non-specific binding analyte interactions as well as prevention of the commonly reported pore clogging^4^. The excellent selectivity and affinity of the biosensor is particularly critical in diagnostics for detecting and identifying rare biomolecules/diseases in clinical samples or other biological fluids. To date, majority of the nanopore studies only work with specific proteins incubating with their specific counterpart in buffer based electrolyte. This contrasts with the real scenario of detecting proteins in samples where non-specific binding to background proteins and environmental/reagent contamination from sample collection is common. With this in mind, we have shown that the method can accurately distinguish between different proteins in a single carrier. We have also illustrated that our detection probes are highly selective and sensitive in buffer as well as unprocessed HS.

Due to the large abundance of selected aptamer sequence and the ability to perform measurement directly from complex biological samples. For example, aptamers already exist that bind specifically and with high affinity to proteins such as α-synuclein oligomers. α-synuclein has a central role in neurodegenerative diseases and particularly parkinsonism, however they are exceptionally challenging to detect with conventional nanopore technology, due to high diffusion rates, heterogeneous surface charge and fast molecular transport. We believe that such measurements can be performed directly from cerebral spinal fluid which would potentially enable a rapid diagnostic tool with single-molecule sensitivity.

## Methods

### Solutions and reagents

λ-DNA which consisted of 12 bases overhangs was purchased from New England Biolabs, UK Catalog #3011 S and all designed aptamer probes (Supplementary Figs. 3 and 4) were obtained from Invitrogen custom oligonucleotides. α-thrombin was obtained from Cambridge Biosciences, UK. AChE and HS (from clotted human whole blood) were purchased from Sigma-Aldrich, UK.

### DNA/aptamer detection probe hybridisation

5 μL of with a stock concentration of λ-DNA 500 μg mL^−1^ and 1 μL of the aptamer probes (see Supplementary Figs. 3 and 4 for sequences) with a stock concentration of 5 μM were mixed in a total volume of 20 µL of 150 mM NaCl, 10 mM MgCl_2_ Tris-EDTA buffer at pH 7.4 followed by 5 mins heating to 95 °C, 10 min annealing to 65 °C and cooling to room temperature for 10 min. The excess aptamer probes were then removed by 100 kDa MWCO Amicon Ultra filter (Millipore, UK) before incubating with targets allowing ~1:1 ratio of the DNA carrier to aptamer probe.

### Nanopipette fabrication

Quartz capillaries (Intracel Ltd, UK) length 75 mm with 0.5 mm filament was placed inside a plasma cleaner to remove any organic contaminants. Nanopipettes were fabricated using a P-2000 laser-based pipette puller (Sutter Instrument, USA). The pipettes used in Figs. 2, 3 and 5 were fabricated from protocol reported^12, 13^. The pipettes used in Fig. 4 were fabricated using a two-line programme: (1) HEAT: 775; FIL: 4; VEL: 30; DEL: 170; PUL: 80, (2) HEAT: 825; FIL: 3; VEL: 20; DEL: 165; PUL: 180 to yield a conductance of 5.8 ± 0.8 nS (mean ± 1 standard deviation). It should be noted that the above parameters are instrument specific and might have slight variations due to local temperature and humidity.

### Ionic current measurement and detection

All nanopore studies were carried out at room temperature and were performed in 100 mM KCl, 10 mM Tris and 1 mM EDTA solution at pH 8 unless otherwise stated. The DNA carrier concentration was 100 pM and protein concentrations ranged from 1.6 nM–4.8 nM for thrombin and 6.8 nM for AChE (100 mM KCl, 10 mM Tris and 1 mM EDTA buffer).

Measurements were carried out with an Axopatch 200B patch clamp amplifier (Molecular Devices, USA). The analyte was placed inside the negatively charged quartz nanopore and the headstage was connected via Ag/AgCl electrodes. Quartz nanopore dimensions were measured by SEM and by ionic conductance indicating nanopore diameters between ~15–40 nm. The signal was filtered using a low pass Bessel filter at either 5 or 10 kHz and digitised with a Digidata 1440A at a sampling rate of 50–100 kHz. Data was processed using a custom written Matlab script.

### Defining the fractional position of the bound protein

The fractional position of the bound protein along the DNA carrier was determined using the following algorithm with custom code written in Matlab. (1) The I-t signal was tracked and subtracted to compensate for any baseline fluctuations. (2) The background noise was determined by fitting a Poisson distribution to the first peak in an all points histogram. (3) A signal of 7 standard deviations above the mean noise level was used to define a threshold, i.e., all signal above this threshold was classified as an event and anything below was discarded. (4) Individual events where then isolated using a peak finding routine whereby parameters such as peak maximum, DNA carrier maximum, total peak width, and peak area could be determined. Event times were determined by using the FWHM of the DNA carrier signal. (5) Once individual events have been classified, each event was analysed for secondary peaks. This was performed both manually and via a multi-step threshold routine. (6) Events associated with possible knot formation and folded DNA are removed from further analysis unless otherwise stated. (7) Finally, sub-peak statistics of the secondary peaks are then extracted (e.g., number of peaks, event time, amplitude, area, and fractional position).

### Data availability

The data that support the findings of this study are available from the corresponding authors upon request.

## Electronic supplementary material

Supplementary Information
