# Supplementary material for: Single molecule multiplexed nanopore protein screening in human serum using aptamer modified DNA carriers
Source: Nat Commun. 2017 Nov 16;8:1552. doi: 10.1038/s41467-017-01584-3 (PMC5691071; doi:10.1038/s41467-017-01584-3)
Supplement: Supplementary file 1 — Supplementary Information [file 41467_2017_1584_MOESM1_ESM.pdf]

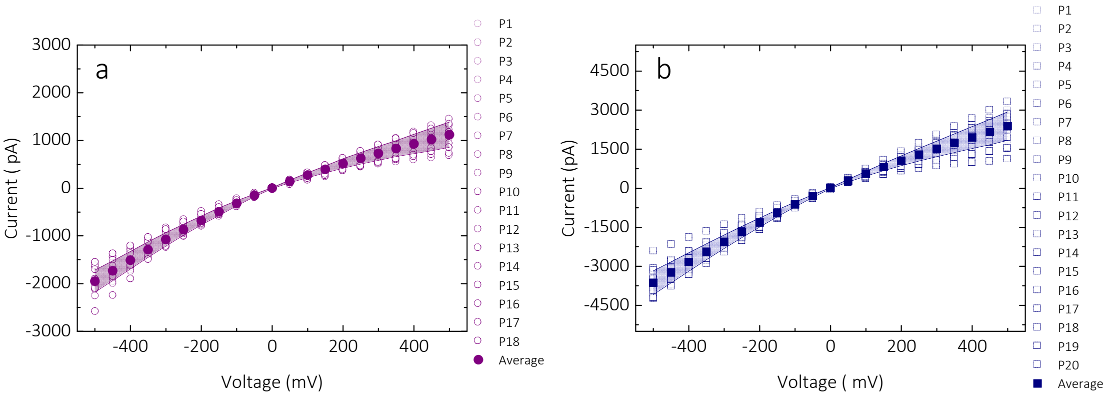

Supplementary Figure 1. Electrical nanopipette characterisation from a total of 38 Quartz nanopores. The quartz nanopores were fabricated with a quartz capillary inserted into a laser-based pipette puller yielding two asymmetric pores. The pores were then characterised electrically and optically. Details of the fabrication protocol can be found in supplementary references 1-5. (a) Current-voltage curves from quartz nanopores with conductance 2.8 ± 0.5 nS (n = 18), used for experiments shown in Figures 2,3, and 5. (b) Current-voltage curves from quartz nanopores with conductance of 5.8 ± 0.8 nS (n = 20) used for experiments shown in Figure 4. All measurements were performed in 0.1 M KCL Tris-EDTA buffer.

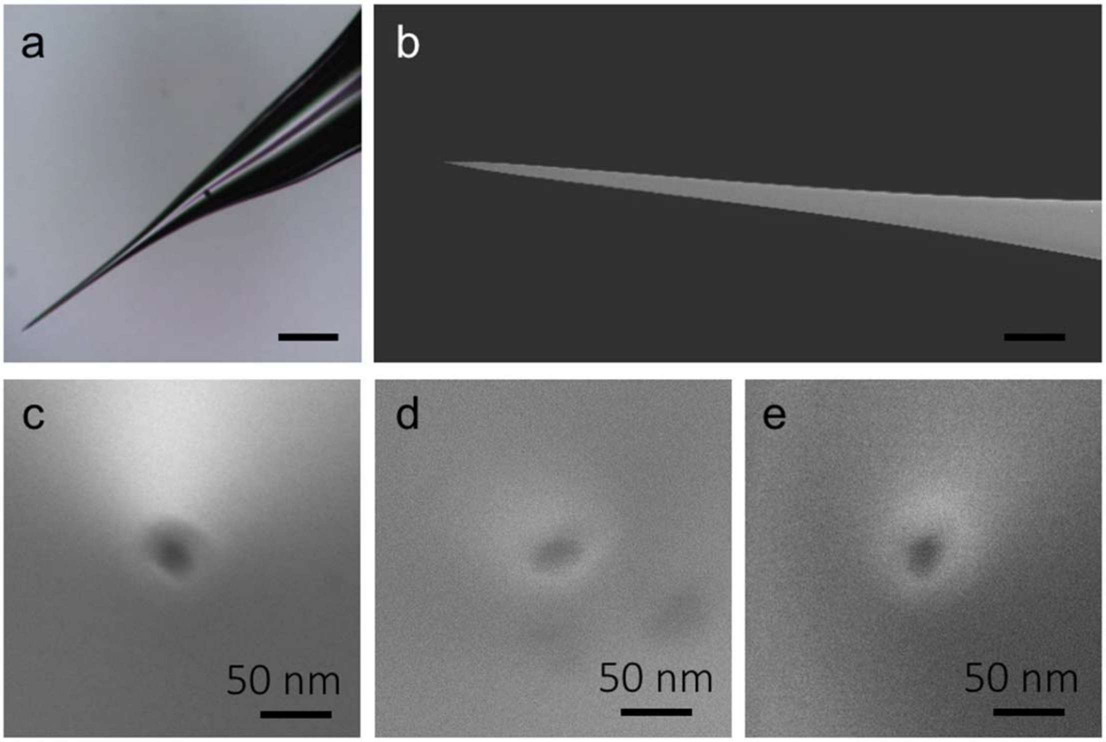

Supplementary Figure 2. Optical and SEM imaging of the nanopipette. (a) Bright field optical image (scale bar 500 µm) and (b) SEM showing the conical geometry and taper length of the quartz nanopipette, scale bar 50 µm. (c-e) Representative SEMs of nanopipettes used in experiments. The average diameters and 1 standard deviation as measured by SEM were 16 ± 2, 19 ± 2 nm, and 16 ± 2 nm (in all cases n=4), respectively. Scale bars are 50 nm.

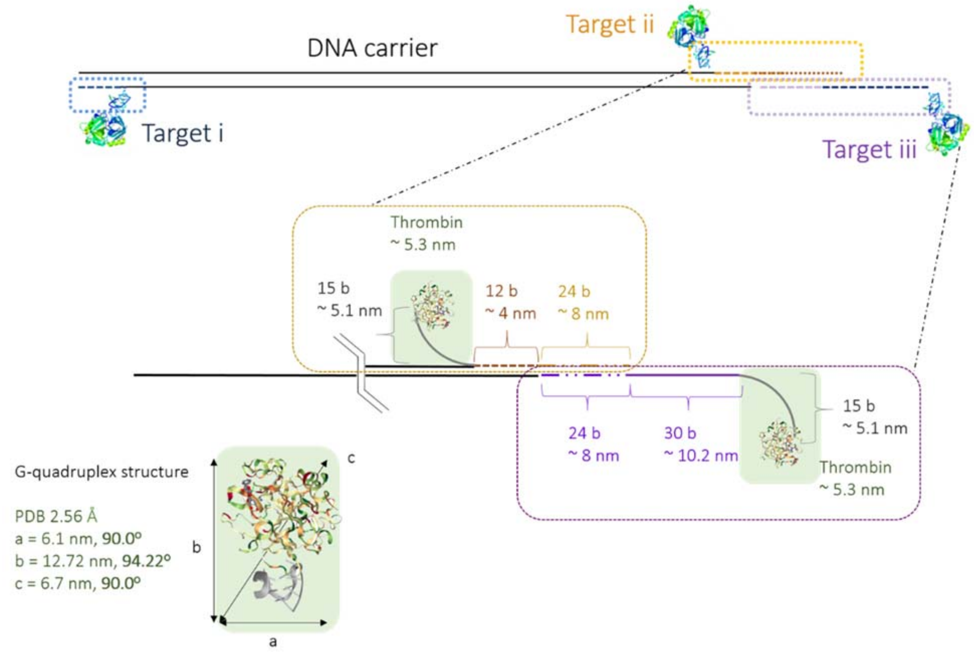

Supplementary Figure 3. Schematic and sequences used for detecting three thrombin protein targets. The sequences used for the modification of the DNA carrier with aptamer probes are as follows: Target i (27 bases): 5’ - *GGTTGG TGTGGTTGG*AGGTCGCCGCCC – 3’, Target ii (51 bases): 5’-*GGTTGGTGTGGTTGG*GGGCGGCGACCTAAGGTGTCGTG CGTAAGTTTTTAA – 3’, Target iii (69 bases): 5’ - *GGTTGGTGTGGTTGG*TTT TTTTTTGTCTTTTTTTTTTTTTCTGTTTTTAAAAACTTACGCACGACACCTT- 3’. In all cases the aptamer sequences are shown is shown in black and underlined.

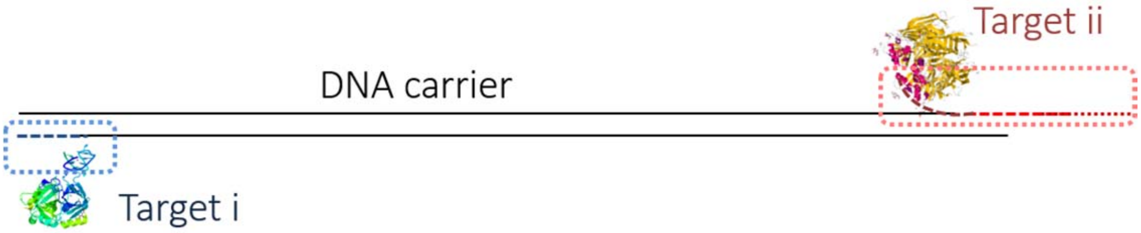

Supplementary Figure 4. Schematic of the designed aptamer detection probes used for thrombin and AChE. The sequences used for the modification of the DNA carrier with aptamer probes specific to thrombin and AChE are as follows: Target i (27 bases, thrombin): 5’ - *GGTTGGTGTGGTTGG*AGGTCGCCGCCC – 3’, Target ii (75 bases, AChE): 5’ - *GGTTGACTGTAGCTCTGGCAGACGTAGTGTGAAGGTACC***GGGCGGCGACCT**AAGG TGTCGTGCGTAAGTTTTTAA – 3’. In all cases the aptamer sequences are shown is shown in black and underlined.

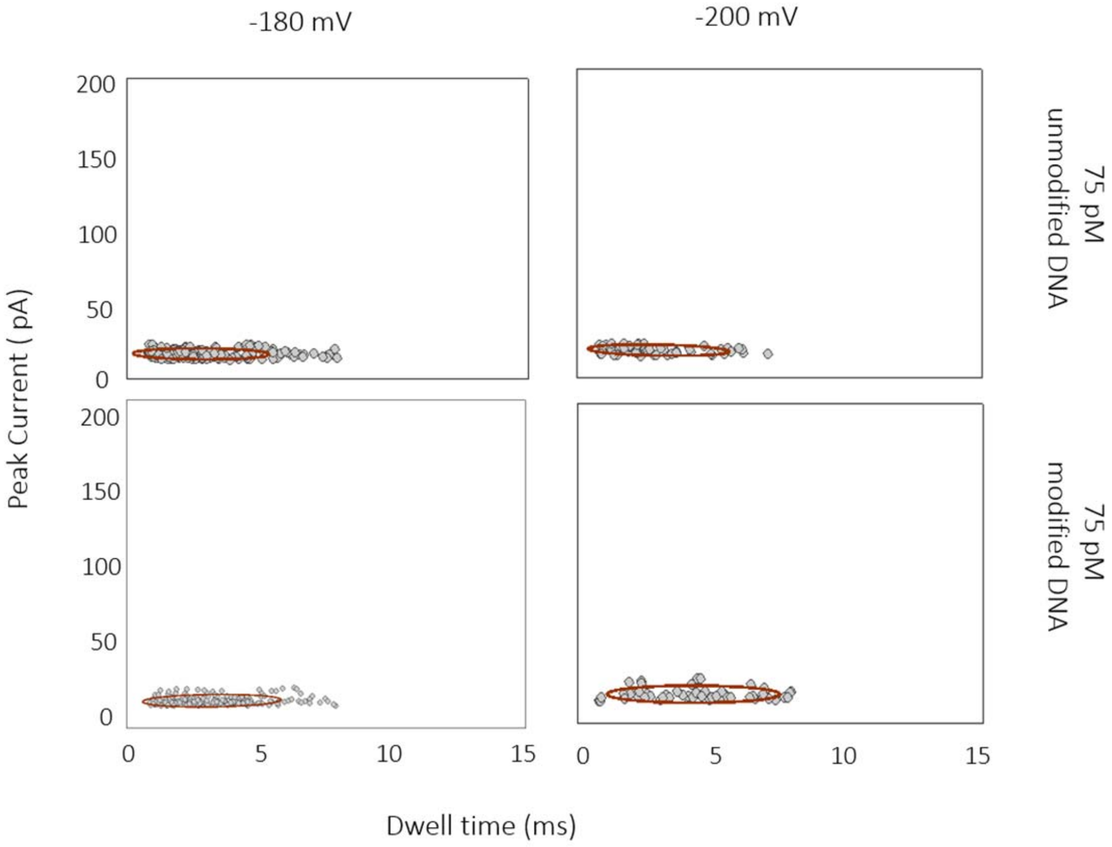

Supplementary Figure 5. Scatter plots for both aptamer modified and unmodified DNA carrier. Negligible change in the current and dwell time distributions were observed when comparing aptamer modified and unmodified DNA at –180 and -200 mV.

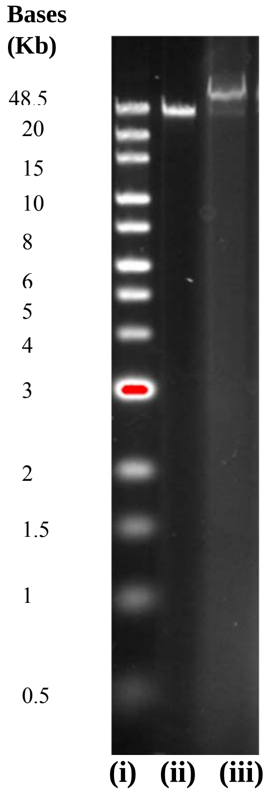

Supplementary Figure 6. Gel electrophoresis image for the unmodified and aptamer modified DNA carrier. From left to right (i) Extended 1 kb DNA ladder (ii) unmodified λ – DNA (iii) aptamer modified λ – DNA. A 0.65% agarose gel was used and separations were obtained at a voltage of 5.7V/ cm for 1 hr in TBE buffer. The gel was then stained with SYBR Gold and imaged under UV light. The modified carrier shows that the majority of the aptamers are bound to the carrier causing a very small shift in the gel.

a

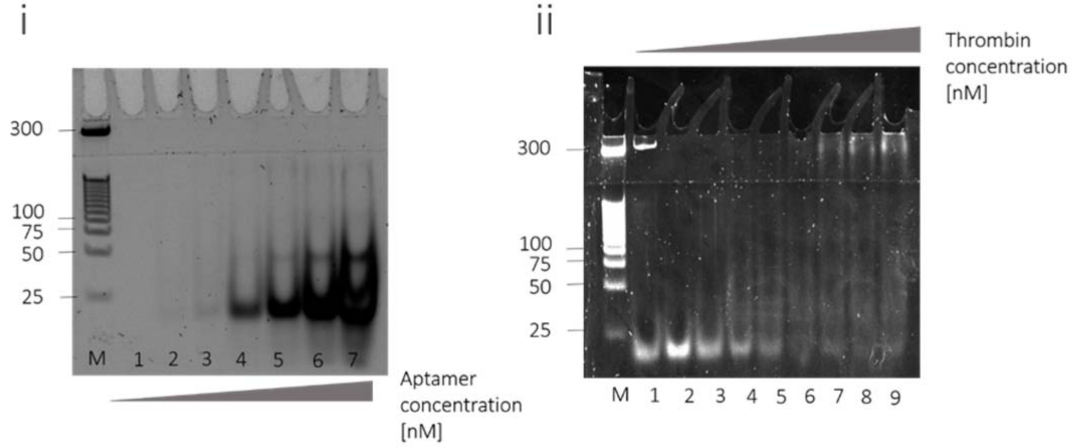

b

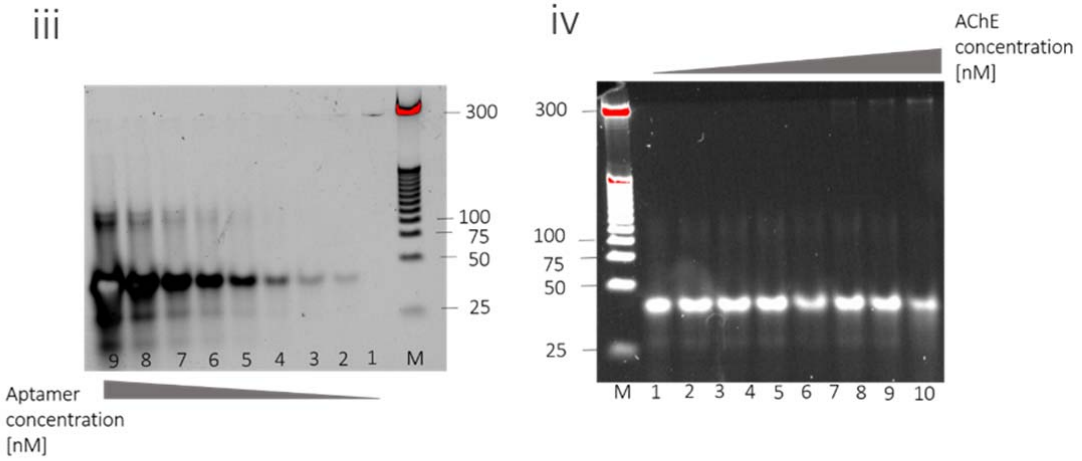

Supplementary Figure 7. Electrophoretic mobility shift assay (EMSA) to confirm the binding of the aptamers to thrombin and AChE targets. All gels shown above include a 25 bp ladder and indicated as M in the gel. (i) Thrombin (750 nM) was incubated with different concentration of modified TBA aptamer (top left) ranging from 0 – 5000 nM in 140 mM NaCl, 20 mM $MgCl_2$, 20 mM PBS buffer pH 7.4 for 45 mins. The 7 lanes on the gel represent the following concentrations of aptamer (from left to right): 0, 50, 100, 250, 500, 1000 and 5000 nM. The samples were then loaded on a 10 % native polyacrylamide cast gel and ran for 75 min at 80V and stained with SYBR gold and imaged under UV. The bottom band showed the unbound aptamer and as the aptamer concentration increases, a gradual shift showing the retardation of mobility by the thrombin binding to the modified aptamer. (ii)

modified TBA (200 nM) was then incubated with different concentration of thrombin in the same binding buffer as (i) for 45 min. The 9 lanes consist of different thrombin concentration from left to right: 0, 250, 500, 1000, 1500, 2500, 5000 and 7500 nM. The samples were then loaded to a 10 % native polyacrylamide cast gels and ran for 75 min at 80V and stained with SYBR gold and imaged. (iii) AChE (660 nM) was incubated with different concentration of modified AChE aptamer ranging from 0 – 5000 nM in 2.7mM KCl, 4 mM $MgCl_2$ PBS buffer pH 7.4 for 1 hour. The 9 lanes consist of different aptamer concentration from (left to right) 5000, 2500, 1000, 500, 250, 100, 50, 25 and 0 nM and placed in a 8% polyacrylamide cast gel and ran at 90 V for 75 min. The gel was then stained and imaged. A very clear band shift is observed as can be seen due to the sluggish moving of the protein-aptamer. (iv) Same format as (ii) however with AChE protein, AChE aptamer was fixed at 100 nM with a range of AChE concentration from left to right 0, 10, 50, 100, 250, 660, 1000, 2000 nM in the same binding buffer and placed in a 8% polyacrylamide gel and ran at 90V for 75 min. The gel was then stained with SYBR gold and imaged.

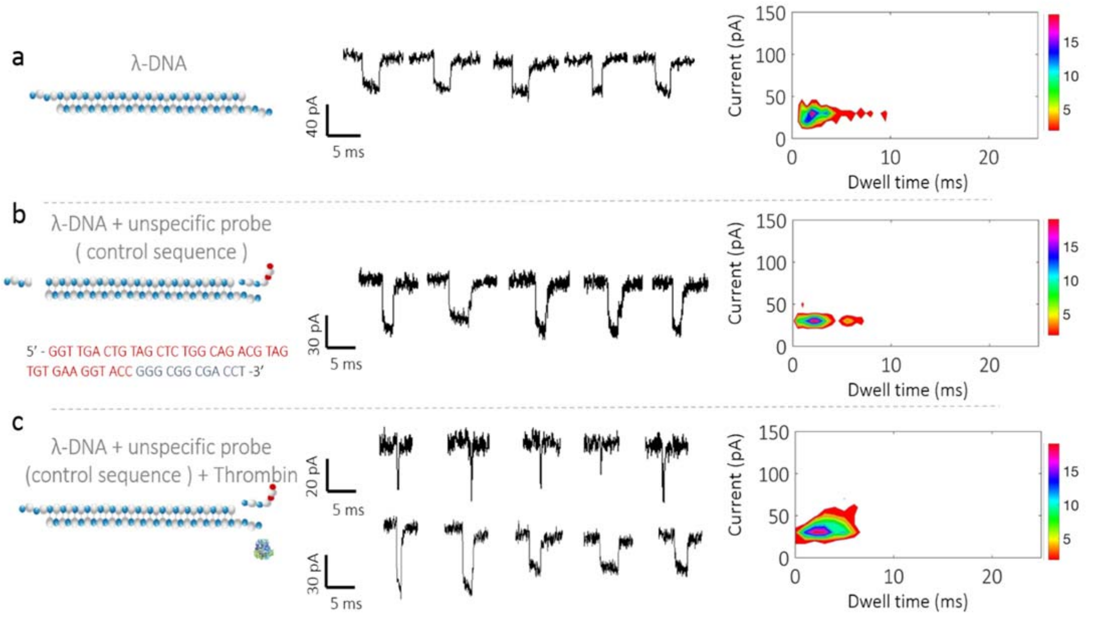

Supplementary Figure 8. Control experiments using non-specific aptamers bound to the DNA carrier. (a) unmodified λ-DNA, (b) aptamer modified λ-DNA and (c) λ-DNA + non-specific probe + thrombin. In all cases the DNA carrier concentration was 100 pM and no sub-peaks were observed (as shown in Figure 2) with exception of folded DNA.

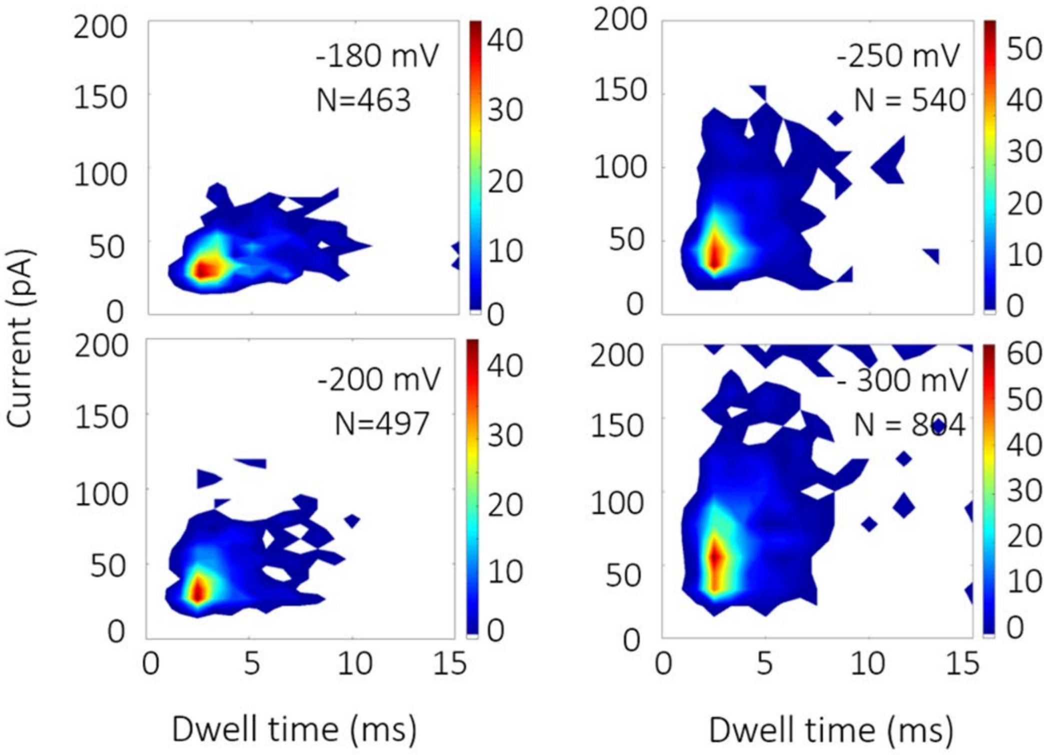

Supplementary Figure 9. Current and dwell time scatter plots for two proteins, thrombin and AChE bound to the same DNA carrier. This corresponding to the data shown in Figure 4.

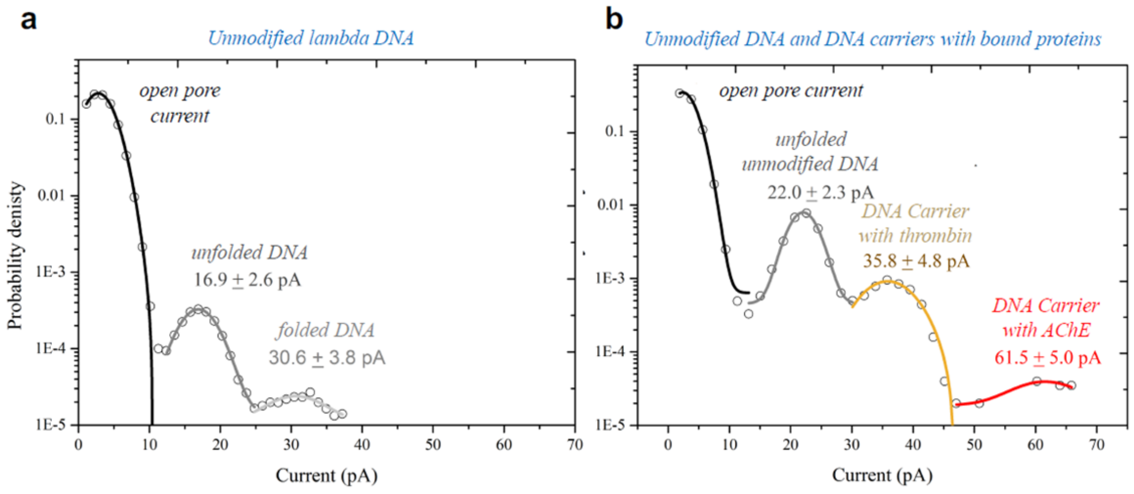

Supplementary Figure 10. Algorithm used for sub-peak thresholding. The method we use to differentiate between different molecular populations is as follows: 1) By using an all points current histogram we fit a Poisson distribution to define the background current. 2) The remaining peaks are fit to Gaussian distributions, and in the case of this data set we use a three-sigma confidence level to differentiate between free and protein bound DNA as shown below. 3) Each individuate translocation is assessed to confirm they exhibit multilevel behaviour. Only translocations with a minimum of 2 steps are defined as originating from a modified DNA carrier with bound protein. 4) Finally, the sub-peaks are analysed to remove the small underlying population due to folded DNA. Examples are shown for (**a**) unmodified λ DNA carrier and (**b**) λ DNA carrier bound to thrombin and AChE proteins. Note that in the example shown, different nanopipettes are used which results in a slight offset between peak amplitudes. As shown above this analysis is performed on a single data file.

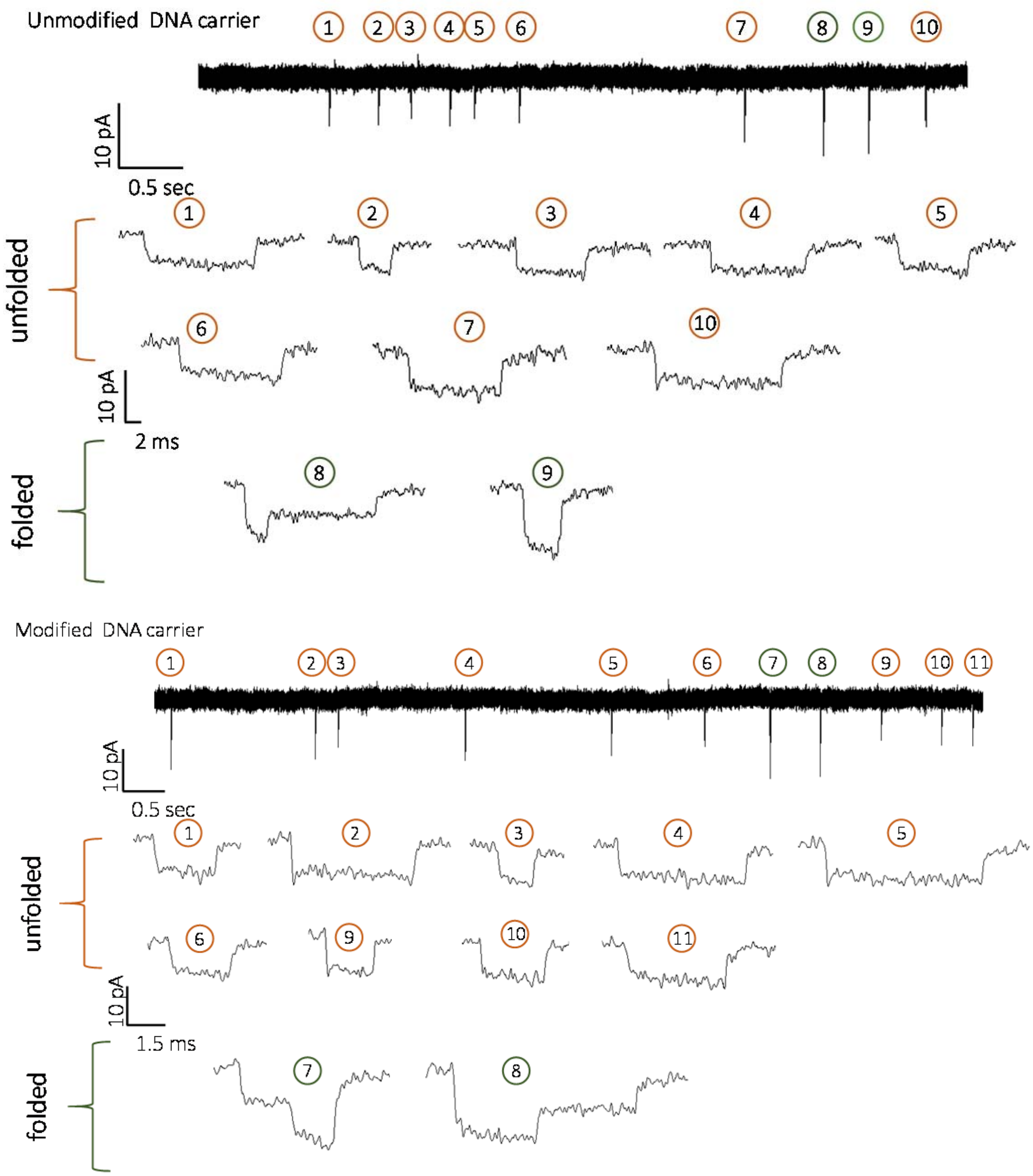

Supplementary Figure 11. Representative examples of unfolded and folded DNA for both the unmodified and aptamer modified DNA carrier. Continuous current-time trace and zoomed individual translocation events for both unmodified and aptamer modified DNA carrier. Events #8 and #9 in top trace # 7 and #8 in the bottom show representative translocation events in folded configuration. On average 10% of all translocation events were observed to have a folded component. The applied voltage was -150 mV. The traces were filtered with a low pass filter with a cut-off frequency of 5 kHz for visualisation.

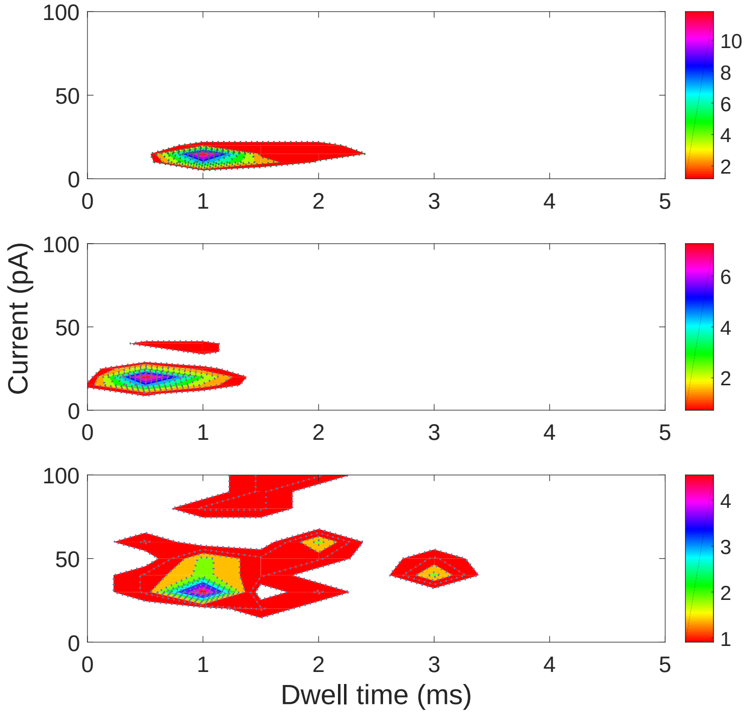

Supplementary Figure 12 |. Sub-peak comparison for thrombin and AChE bound to a DNA carrier. Scatter plots are shown for thrombin, AChE, and and the sub-peak associated with folded DNA for reference. All measurements were taken at -200 mV. There is less than 1% overlap between AChE and folded DNA based on the sub-peak amplitude and dwell time and less than 10 % overlap between thrombin and folded DNA.

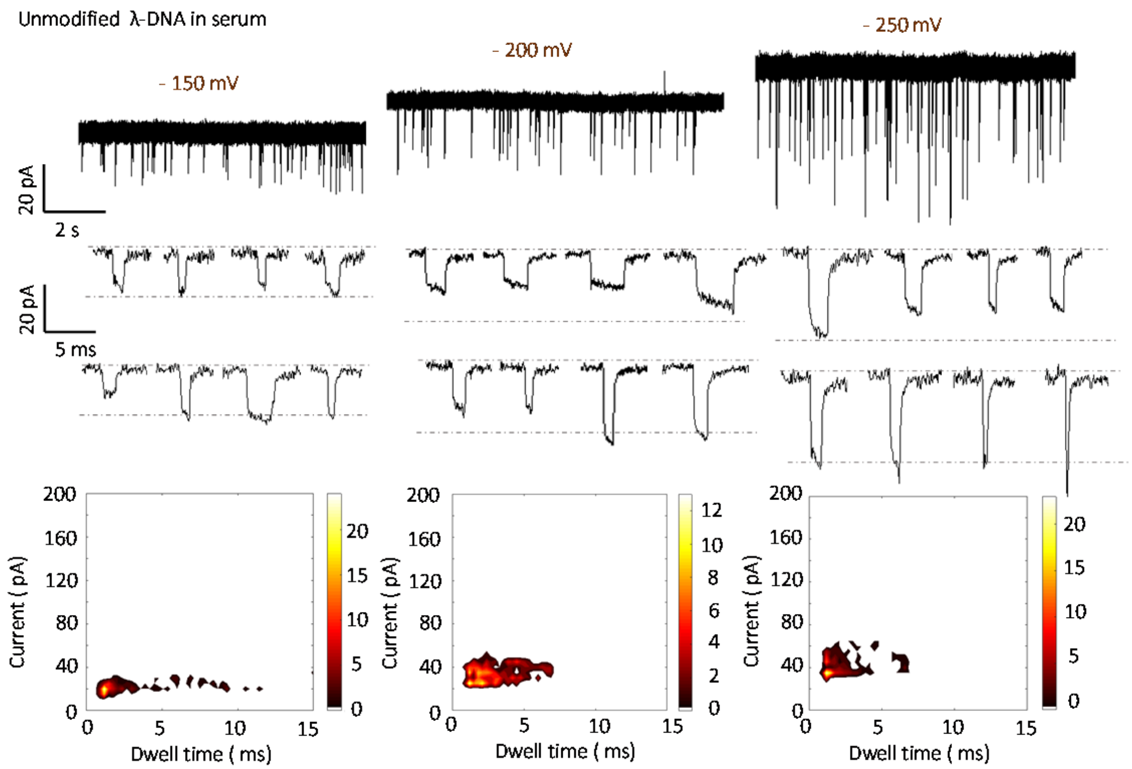

Supplementary Figure 13. Control experiments for unmodified DNA carrier in serum. Examples of typical current-voltage traces along with voltage dependant scatter plots are shown for -150, -200 and -250 mV respectively. In all cases statistics are similar to detection of unmodified DNA in buffer alone.

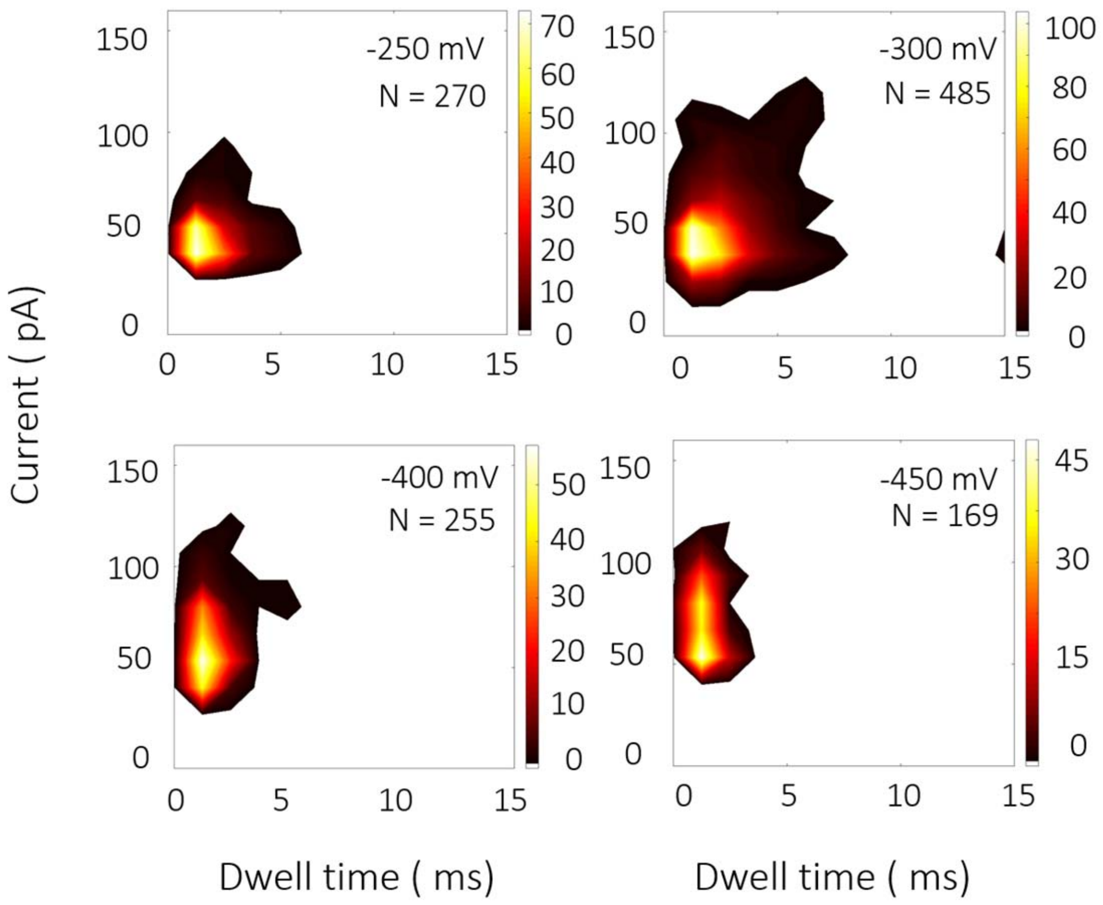

Supplementary Figure 14. Current and dwell time scatter plots corresponding to the data shown in Figure 5. DNA concentration was 100 pM and voltage was varied between -250 to 450 mV.

Supplementary Table 1. Analysis summary of possible DNA knot formation.

**0.1M KCl**	Voltage (mV)	Possible knots (%)	Multiple knots (%)	Low pass filter cut-off (kHz)
NP1	-180	0.2	0	10
	-200	0.8	0	10
NP2	-180	0.4	0.4	10
	-200	0	0	10
NP3	-180	0.8	0	10
	-200	0.4	0.5	10

To quantify potential false positives, all translocation data for unmodified lambda DNA and aptamer modified DNA was analysed for possible knots. We classify all secondary "spikes" below 80 μs to be a possible knot.[6] Note that under the conditions used in this manuscript knots are expected to be transported quicker and for the most part go unobserved. A summary is shown in the table along with 2 examples of possible knots.

Supplementary References

1. Gong X*, et al.* Label-free in-flow detection of single DNA molecules using glass nanopipettes. *Analytical chemistry* **86**, 835-841 (2014).

2. Ivanov AP, Actis P, Jönsson P, Klenerman D, Korchev Y, Edel JB. On-Demand Delivery of Single DNA Molecules Using Nanopipets. *ACS Nano* **9**, 3587-3595 (2015).

3. Sze JY, Kumar S, Ivanov AP, Oh SH, Edel JB. Fine tuning of nanopipettes using atomic layer deposition for single molecule sensing. *Analyst* **140**, 4828-4834 (2015).

4. Crick CR, Sze JYY, Rosillo-Lopez M, Salzmann CG, Edel JB. Selectively Sized Graphene-Based Nanopores for in Situ Single Molecule Sensing. *ACS Applied Materials & Interfaces* **7**, 18188-18194 (2015).

5. Freedman KJ, Otto LM, Ivanov AP, Barik A, Oh S-H, Edel JB. Nanopore sensing at ultra-low concentrations using single-molecule dielectrophoretic trapping. *Nature communications* **7**, 10217 (2016).

6. Plesa C*, et al.* Direct observation of DNA knots using a solid-state nanopore. *Nat Nanotechnol* **11**, 1093-1097 (2016).
